# Combined effects of nutritional, biochemical and environmental stimuli on growth performance and fatty acid composition of gilthead sea bream (*Sparus aurata*)

**DOI:** 10.1371/journal.pone.0216611

**Published:** 2019-05-14

**Authors:** Claudia Torno, Stefanie Staats, Anna Fickler, Sonia de Pascual-Teresa, María Soledad Izquierdo, Gerald Rimbach, Carsten Schulz

**Affiliations:** 1 GMA—Gesellschaft für Marine Aquakultur mbH, Büsum, Germany; 2 Marine Aquaculture Research Group, Institute of Animal Breeding and Husbandry, Faculty of Agricultural and Nutritional Sciences, University of Kiel, Kiel, Germany; 3 Food Science Research Group, Institute of Human Nutrition and Food Science, Faculty of Agricultural and Nutritional Sciences, University of Kiel, Kiel, Germany; 4 Department of Metabolism and Nutrition, Institute of Food Science, Food Technology and Nutrition (ICTAN–CSIC), Madrid, Spain; 5 Grupo de Investigación en Acuicultura (GIA), Instituto Universitario Ecoaqua, Universidad de Las Palmas de Gran Canaria, Telde, Las Palmas, Canary Islands, Spain; University of Illinois, UNITED STATES

## Abstract

The reliance of the aquafeed industry on marine resources has to be reduced by innovative approaches in fish nutrition. Thus, a three-factorial approach (fish oil reduced diet, phytochemical genistein, and temperature reduction) was chosen to investigate the interaction of effects on growth performance and tissue omega-3 long chain polyunsaturated fatty acid (LC-PUFA) levels in juvenile sea bream (*Sparus aurata*, 12.5 ± 2.2 g). Genistein is a phytoestrogen with estrogen-like activity and thus LC-PUFA increasing potential. A decrease in the rearing temperature was chosen based on the positive effects of low temperature on fish lipid quality. The experimental diets were reduced in marine ingredients and had a fish oil content of either 6% dry matter (DM; F6: positive control) or 2% DM (F2: negative control) and were administered in the plain variant or with inclusion of 0.15% DM genistein (F6 + G and F2 + G). The feeding trial was performed simultaneously at 23°C and 19°C. The results indicated that solely temperature had a significant effect on growth performance and whole body nutrient composition of sea bream. Nevertheless, the interaction of all three factors significantly affected the fatty acid compositions of liver and fillet tissue. Most importantly, they led to a significant increase by 4.3% of fillet LC-PUFA content in sea bream fed with the diet F6 + G in comparison to control fish fed diet F6, when both groups were held at 19°C. It is hypothesized that genistein can act via estrogen-like as well as other mechanisms and that the dietary LC-PUFA content may impact its mode of action. Temperature most likely exhibited its effects indirectly via altered growth rates and metabolism. Although effects of all three factors and of genistein in particular were only marginal, they highlight a possibility to utilize the genetic capacity of sea bream to improve tissue lipid quality.

## Introduction

In order to reduce the reliance on marine resources from capture fisheries, namely fish meal and fish oil, the aquafeed industry has turned to plant products. Often, the reduced costs and improved sustainability of plant ingredients advance their inclusion in diets for farmed fish [1,2]. Fish farmed on diets low in fish meal and fish oil can be subject to reduced lipid quality in terms of low amounts of omega-3 (n-3) long chain polyunsaturated fatty acids (LC-PUFAs) like eicosapentaenoic acid (EPA; 20:5n-3) and docosahexaenoic acid (DHA; 22:6n-3) [3,4]. Besides potential effects of the reduced n-3 LC-PUFAs on the fish’s health status, physiological functions, and development [5,6], the nutritional benefits of fish for human nutrition might be decreased. Such reduced lipid quality is especially distinctive in carnivorous marine fish that depend on their diet as a source for n-3 LC-PUFAs. The *in vivo* hepatic fatty acid synthesis converting the precursor fatty acid α-linolenic acid (ALA; 18:3n-3) to EPA and DHA is disrupted in marine fish [6]. Although the genetic capacity is present, the functionality of certain enzymes is impaired [7–10]. For these reasons, suitable fish oil replacements like novel EPA and DHA rich sources [11–13], oils rich in functional fatty acids like stearidonic acid [14], or strategies improving the efficiency of fish oil utilization need to be investigated. The latter might be realized via feeding strategies like alternate feeding of fish oil and vegetable oil [15,16] and parental nutritional programming [17]. Another alternative might be the use of functional and bioactive food supplements that interact with the lipid metabolism of fish, protect fatty acids from oxidation, or stimulate the *in vivo* synthesis of EPA and DHA from ALA [18–22].

One phytochemical with potential effects on lipid quality is genistein, an isoflavonoid compound found in Leguminosae [23]. Its structural similarity to estrogen is expected to promote its bioactivity and health benefits [24–28]. There is good evidence that genistein affects the lipid metabolism in different fish species and alters plasma triglyceride and cholesterol levels as well as overall growth rates [29–31]. Furthermore, genistein could lead to an improvement of the lipid quality via enhancing the expression of genes involved in fatty acid catabolism and synthesis, like the peroxisome proliferator-activated receptor α (PPARα) and its target genes [21,25,32].

Besides nutritional factors, also environmental factors like temperature can influence the lipid metabolism and thus lipid quality of fish [5]. Several studies demonstrated that changes in temperature can affect the ratio of saturated to monounsaturated fatty acids and influence the fatty acid synthesis in fish [5,33,34]. Skalli et al. [35] demonstrated that low water temperatures led to an increased amount of n-3 LC-PUFAs in different tissues of European sea bass (*Dicentrarchus labrax*).

Though research has provided impressive information in past years, a promising approach to guarantee high lipid quality of marine fish farmed with aquafeeds highly reduced in marine ingredients is still lacking. We hypothesize that the combination of different stimuli might be most effective to improve the fatty acid composition with regard to EPA and DHA in gilthead sea bream (*Sparus aurata*): nutritional stimuli (dietary EPA and DHA level), biochemical stimuli (the bioactive substance genistein), and environmental stimuli (low temperature).

To evaluate our hypothesis, we conducted an experimental trial with sea bream to investigate the effects of holding temperature (19°C and 23°C) and low-fish oil diets supplemented with dietary genistein on (1) growth, (2) performance parameters, (3) whole body nutrient composition, and (4) fatty acid composition of the whole body, liver and fillet tissue of juvenile sea bream.

## Materials and methods

### Experimental diets

Four different isonitrogenous and isoenergetic experimental diets were formulated as shown in Table 1. The diets were reduced in animal protein components (5% dry matter (DM) fish meal and 7% DM blood meal) and consisted mainly of plant protein sources (soy protein concentrate, corn gluten, wheat gluten, and rapeseed expeller). The diets only differed in the composition of the oil fraction with varying amounts of fish and vegetable oils. Diet F6 served as positive control and had 6% DM fish oil and 3% DM of vegetable oils (linseed oil, rapeseed oil, and palm oil). Diet F2 was regarded as negative control and had a reduced fish oil content of 2% DM and 7% DM of vegetable oils (linseed and rapeseed oil). Conditioned by the difference in the fish oil inclusion, the dietary fatty acid composition differed considerably (Table 2). The control diet F6 contained EPA and DHA amounts that met the recommendations for sea bream (EPA + DHA: 0.9% of dry diet when DHA/EPA = 1 [36,37]). The amounts of EPA and DHA were reduced below the recommendations in the negative control diet F2 (0.37% of dry diet, Table 2). The experimental diets F6 + G and F2 + G were equal to diets F6 and F2, respectively, with addition of 0.15% DM of diet genistein (+G; 5,7-dihydroxy-3-(4-hydroxyphenyl)-(4H)-benzopyran-4-one, purity ≥ 98%, Chemos GmbH & Co. KG, Regenstauf, Germany). Furthermore, the amount of ALA was equal in all diets. All diets were formulated to meet the macronutrient and essential amino acid requirements of gilthead sea bream according to Peres and Oliva-Teles [38] and Wilson [39]. The diets were produced in 3- and 4-mm pellets with a feed press L14-175 (Amandus Kahl, Reinbek, Germany).

**Table 1:** Ingredients and nutrient composition (percentage of dry matter: % DM) of the experimental diets. F6 and F2 are the control diets with 6% and 2% DM fish oil, respectively. +G indicates the supplementation of the control diets with 0.15% DM genistein.

*Ingredients [% DM]*	F6	F2	F6 + G	F2 + G
Fish meal (*Clupea sp*.) ^1^	5.00	5.00	5.00	5.00
Soybean concentrate (HP 300) ^2^	19.00	19.00	19.00	19.00
Blood meal ^3^	7.00	7.00	7.00	7.00
Corn gluten ^2^	18.00	18.00	18.00	18.00
Wheat gluten ^4^	12.50	12.50	12.50	12.50
Rapeseed expeller ^5^	8.00	8.00	8.00	8.00
Wheat starch ^4^	10.96	10.96	10.96	10.96
Fish oil ^1^	6.00	2.00	6.00	2.00
Linseed oil ^6^	2.00	2.08	2.00	2.08
Rapeseed oil ^7^	0.10	1.65	0.10	1.65
Palm oil ^8^	0.90	3.27	0.90	3.27
Vitamin Mineral premix ^9^	0.50	0.50	0.50	0.50
Methionine ^9^	0.85	0.85	0.85	0.85
Lysine ^10^	1.19	1.19	1.19	1.19
Dicalciumphosphate ^11^	2.08	2.08	2.08	2.08
Inositol ^12^	0.02	0.02	0.02	0.02
Cholin chloride ^13^	0.13	0.13	0.13	0.13
Cholesterol ^12^	0.11	0.11	0.11	0.11
Lecithin ^12^	2.53	2.53	2.53	2.53
α-Cellulose ^14^	2.00	2.00	2.00	2.00
Inert filler ^15^	1.13	1.13	1.13	1.13
Genistein (G) ^16^			0.15	0.15
*Nutrient composition [% DM]*				
Dry matter	90.7	90.9	90.5	91.1
Crude ash	7.1	6.9	7.0	7.1
Crude protein	49.3	49.7	50.1	50.0
Crude lipid	14.5	14.6	14.5	14.7
Total carbohydrates	29.1	28.8	28.4	28.3
Gross energy [MJ kg^-1^ DM]	22.89	22.83	22.86	22.86

^1^ Vereinigte Fischmehlwerke Cuxhaven GmbH & Co. KG, Cuxhaven, Germany

^2^ EURODUNA Rohstoffe GmbH, Barmstedt, Germany

^3^ Sonac, Son, Netherlands

^4^ KRÖNER STÄRKE GmbH, Ibbenbüren, Germany

^5^ Stöfen Landhandel, Wesselburen, Germany

^6^ Makana Produktion und Vertrieb GmbH, Offenbach a.d.Queich, Germany

^7^ Different food stores, Büsum, Germany

^8^ EFG Elbe Fetthandel GmbH, Geesthacht, Germany

^9^ Emsland-Aller Aqua GmbH, Golßen, Germany

^10^ Biolys: Evonik Industries AG, Essen, Germany

^11^ J. Rettenmaier und Söhne GmbH und Co. KG, Rosenberg, Germany

^12^ Roth GmbH, Karlsruhe, Germany

^13^ Sigma Aldrich, St. Louis, USA

^14^ Mikro-Technik GmbH & Co. KG, Bürgstadt/Main, Germany

^15^ Bentonite: Del Lago Bentonite, Castiglioni Pes y Cía., Buenos Aires, Argentina

^16^ CHEMOS GmbH & Co. KG, Regenstauf, Germany.

**Table 2:** Fatty acid composition (in % of total fatty acid methyl esters (FAMEs) and % of dry matter of diet (% DM)) of the experimental diets. F6 and F2 are the control diets containing 6% and 2% DM fish oil, respectively. +G indicates the supplementation of the control diets with 0.15% DM genistein.

*% of FAMEs*	F6	F2	F6 + G	F2 + G
14:0	2.66	1.31	2.60	1.23
16:0	19.29	27.82	19.37	27.39
18:0	3.60	3.74	3.57	3.79
∑ SFA ^1^	25.55	32.87	25.53	32.42
16:1n-7	2.83	1.16	2.62	1.04
18:1n-7c	2.51	1.90	2.43	1.91
18:1n-9c	24.54	24.72	23.72	23.48
∑ MUFA ^2^	29.88	27.78	28.77	26.43
18:2n-6c (LA) ^3^	24.80	24.19	25.76	26.76
18:3n-3 (ALA) ^4^	11.77	12.11	12.47	11.71
18:3n-6	0.15	0.05	0.11	0.06
20:5n-3 (EPA) ^5^	3.13	1.17	3.03	0.89
22:5n-3	0.77	0.26	0.65	0.24
22:6n-3 (DHA) ^6^	3.89	1.58	3.63	1.50
∑ PUFA ^7^	44.52	39.35	45.65	41.16
EPA + DHA	7.02	2.75	6.66	2.39
DHA / EPA	1.24	1.35	0.83	0.59
ALA / LA	0.47	0.50	0.48	0.44
EPA + DHA % DM ^8^	0.95	0.37	0.90	0.33

The standard used for identification of individual FAMEs consisted of all 12 FAMEs shown here and C14:1n-5, which was not detected in the diet samples.

^1^ ∑ SFA is the sum of saturated fatty acids

^2^ ∑ MUFA is the sum of monounsaturated fatty acids

^3^ LA: Linoleic acid

^4^ ALA: α-Linolenic acid

^5^ EPA: Eicosapentaenoic acid

^6^ DHA: Docosahexaenoic acid

^7^ ∑ PUFA is the sum of n-3 and n-6 polyunsaturated fatty acids

^8^ The determination of EPA + DHA % DM of diet was done using the internal standard 13:0 methyl ester and amount of lipid measured in the diet (Table 1).

### Experimental setup

The experimental trial was conducted at the facilities of the Grupo de Investigación en Acuicultura (GIA), Universidad de Las Palmas de Gran Canaria (ULPGC), Telde, Las Palmas, Canary Islands, Spain. In order to realize a simultaneous diet- and temperature-challenge, two experimental setups were used: (I) a flow-through system with 12 tanks for the 23°C temperature challenge and (II) four temperature-controlled small part-recirculating systems equipped with a total of 12 tanks for the 19°C temperature challenge.

(I) The flow-through system had a photoperiod of 12 h light (natural light) during the whole adaptation and experimental period. The cylindrical fiberglass tanks (500 L) were supplied with continuously aerated and filtered seawater (37‰ salinity) at a rate of 600 L h^-1^. The water parameters were determined daily (temperature, oxygen, and pH (pH-Meter Basic 20+, CRISON, Hach Lange Spain, Barcelona, Spain)) or every second day (Ammonia and Nitrite (Royal Ammonia Professional Test and Royal Nitrite Professional Test, Royal Nature, Nesher, Israel)) and were as follows: 22.6 ± 0.6°C, O_2_: 6.2 ± 0.3 mg L^-1^, pH: 8.22, NH_4_: < 0.15, NO_2_: < 0.1.

(II) The part-recirculating systems were kept at a photoperiod of 12 h light (artificial light). Each system had a water volume of 3000 L and was equipped with a sand filter (0.4–1.2 mm), a bio filter, a protein skimmer, and a cooling unit. A single part-recirculating system had three cylindrical fiberglass tanks (500 L) that were supplied with temperature controlled and continuously aerated seawater (37‰ salinity) at a rate of 600 L h^-1^. The water quality parameters were measured as described above and did not differ between the RAS: 19.0 ± 1.5°C, O_2_: 7.1 ± 0.3 mg L^-1^, pH: 8.06, NH_4_: < 1, and NO_2_: < 0.5.

Juvenile gilthead sea bream (600 fish, offspring from brood stock of GIA-ULPGC, initial body weight: 12.5 ± 2.2 g) were acclimated in the flow-through system for 2 weeks. Afterwards, all sea bream were randomly and equally distributed among all 24 tanks of both setups, 25 individuals per tank. During the first week of the experimental period, the part-recirculating systems were continuously cooled down from 23°C to 19°C. During the whole experimental period of 8 weeks, feeding of fish until apparent satiation occurred manually three times per day. The daily administered feed ratios were determined to calculate the daily feed intake (DFI).

All experiments were carried out according to the EU Directive 2010/63/EU for animal experiments and approved by the Ministry of Energy, Agriculture, the Environment, Nature and Digitalization (MELUND), Kiel, Germany (approved on 15^th^ October 2014; project number: V244-7224.121.9–34), as well as by the ethics committee from AQUAEXCEL^2020^.

### Sampling

Gilthead sea bream tissue samples were taken one day prior to the beginning of the feeding trial (initial sample, day 0) and at the end of the trial (day 56). At day 0, a total of seven fish were sacrificed (pooled in one sample) and stored at -80°C for the determination of the whole body nutrient and FA composition. Additionally, for the collection of liver and fillet tissue samples, six sea bream were sacrificed and dissected. The weight (± 0.01 g) of the whole liver was taken for the determination of the Hepatosomatic index (HSI, see formula 1). Afterwards, the livers of all six individuals were pooled in one sample and immediately frozen at -80°C for the determination of the liver fatty acid composition. The pooled fillet sample, consisting of six fillets from the left sides of the six sea bream, were frozen at -80°C for the determination of the fillet fatty acid composition. Comparable samples as described for the initial sampling were taken during the final sampling at day 56. A total of 10 fish per tank were sacrificed: five for the whole body samples (nutrient and fatty acid composition) and five for liver and fillet samples (fatty acid composition), allowing the analysis of fatty acids and nutrients in three samples per treatment, namely one per tank.

The growth performance and nutrient utilization parameters were determined by weighing (± 0.1 g) and measuring (± 0.1 cm) each individual sea bream at day 0 and day 56. The initial and final body weight (IBW and FBW, respectively), specific growth rate (SGR), feed conversion ratio (FCR), protein efficiency ratio (PER), protein productive value (PPV), and Fulton condition factor (FCF) were calculated according to the formulas 2–6.

$$HSI\;[\%]=\frac{liver\;weight\;[g]}{FBW\;[g]}\times 100$$ (1)

$$SGR\;[\%\;d^{-1}]=\frac{\left[ln\;(FBW)-ln\;(IBW)\right]}{feeding\;day}\times 100$$ (2)

$$FCR=\frac{feed\;intake\;[g]}{weight\;gain\;[g]}$$ (3)

$$PER=\frac{weight\;gain\;[g]}{protein\;intake\;[g]}$$ (4)

$$PPV\;[\%]=\frac{\left(final\;body\;protein\;[g]\times FBW\;[g])-(initial\;body\;protein\;[g]\times IBW\;[g]\right)}{protein\;intake\;[g]}\times 100$$ (5)

$$FCF=100\times (FBW[g]\times {final\;body\;length\;[cm]}^{-2})$$ (6)

### Nutrient composition analysis

The nutrient composition analyses of the diets and the whole body homogenates of the gilthead sea bream were done according to EU guideline (EC) 152/2009 [40]. First, the frozen whole body samples were lyophilized (Alpha 1–2 LDplus and Alpha 1–4 LSC, Martin Christ Gefriertrocknungsanlagen GmbH, Osterode am Harz, Germany) and homogenized in a cutting mill (GM 200, Retsch, Haan, Germany). The diets were homogenized with a mortar and pestle. The analyses of nutrients and gross energy were performed as described in Torno et al. [41]. Total carbohydrates were calculated according to the following formula.

Totalcarbohydrates=1000−(crudeprotein+crudelipid+crudeash) (7)

### Lipid extraction and fatty acid composition analysis

The total lipids from the whole body homogenates, liver samples, fillet samples, and diet samples were extracted according to Folch et al. [42] with modifications as in Torno et al. [22]. Samples were neutralized using potassium hydroxide (0.1 M) followed by the addition of the Folch reagent and subsequent centrifugation for 10 min at 2000× *g* to isolate the fatty acid methyl esters (FAMEs). The organic phase was collected to perform a second extraction with potassium hydroxide and the Folch reagent. After subsequent centrifugation (5 min at 2000× *g*) and drying of samples under a N_2_ flux, the re-dissolved FAME samples were injected into a 7820A Agilent gas chromatograph with flame ionization detector (GC-FID; Agilent Technologies, Santa Clara, CA, USA). A FAME-standard was used for the identification of the retention times of the individual FAMEs. The fatty acid composition was calculated as percentage of single FAME relative to total FAMEs. The internal standard 13:0 methyl ester was used to calculate FAs as % DM of diet.

### Statistical analysis

All statistical analyses were performed using R (version 3.1.3) with an RStudio interface. The packages *gdata*, *multcomp*, *gplots*, *nparcomp*, *nlme*, *piecewiseSEM*, *SimComp*, and *car* were used for the graphical and statistical analysis.

The data evaluation started with the definition of an appropriate statistical model based on a graphical residual analysis of the data and Levene’s test to test for homoscedasticity of variances: (1) statistical model based on generalized least squares (*gls*) for normally distributed and heteroscedastic data (PER, FCR, crude protein, and dry matter) and (2) linear model (*lm*) for normally distributed and homoscedastic data (DFI, FBW, FCF, HSI, SGR, IBW, PPV, crude ash, crude fat, gross energy, and fatty acid composition). Both models included the levels of dietary fish oil content (6% DM and 2% DM), supplement (None, + G), and temperature (19°C, 23°C), as well as their respective interaction terms as fixed factors. The analysis of variances (ANOVA) was followed by an appropriate post-hoc test: (1) ANOVA based on *gls* was followed by multiple contrast tests for heteroscedastic data according to Hasler and Hothorn [43]; (2) ANOVA based on *lm* was followed by multiple contrast tests according to Schaarschmidt and Vaas [44].

## Results

### Growth performance and whole body nutrient composition are mainly affected by holding temperature

Throughout the experimental period, all sea bream exhibited good growth. The growth and performance of sea bream was almost unaffected by the dietary treatment and mainly affected by the holding temperature (Tables 3 and 4). The sea bream held at 19°C had an average 2.3 fold increase in body weight and the fish held at 23°C had an average 3.4 fold increase (Table 3). The SGR was affected by the interaction of all three factors: dietary fish oil level, dietary genistein, and holding temperature (Table 4), although the statistical output is partly based on minor differences between single values. The SGR was significantly higher when fish were held at 23°C in comparison to 19°C, but only slightly increased when fish were fed the F6-based diets in comparison to the F2-based diets (p < 0.05, Table 3, indicated by *, a, b and m, n). Dietary genistein led to a slightly but significantly increased SGR when fish were held at 23°C and fed the diet F6 + G in comparison to the diet F6 (p < 0.05, Table 3, indicated by A, B). The DFI was significantly higher when the sea bream were held at 23°C in comparison to 19°C, irrespective of the dietary treatment (p < 0.05, Table 3, indicated by *). The FCR, PER and PPV did not differ between the feeding groups nor between the two temperatures. The higher holding temperature of 23°C led to significantly lower HSI values and significantly higher FCF values, irrespective of the dietary treatment (p < 0.05, Table 3, indicated by *).

**Table 3:** Growth performance, nutrient utilization, and final whole body nutrient composition (percentage of wet weight (% wet weight) and MJ kg^-1^ wet weight) of gilthead sea bream fed with the experimental diets for 8 weeks and held at two different temperatures, 19°C and 23°C. F6 and F2 indicate the feeding with the control diets containing 6% and 2% DM fish oil, respectively. +G indicates feeding the diets supplemented with 0.15% DM genistein.

	19°C				23°C				Comparison between 19 and 23°C			
	F6	F2	F6 + G	F2 + G	F6	F2	F6 + G	F2 + G	F6	F2	F6 + G	F2 + G
IBW ^1^	12.4 ± 0.3	12.5 ± 0.3	12.7 ± 0.3	12.6 ± 0.1	12.6 ± 0.2	12.2 ± 0.2	12.3 ± 0.3	12.4 ± 0.1				
FBW ^2^	29.7 ± 0.9 ^a^	27.0 ± 1.7 ^b^	31.0 ± 0.3 ^(m)^	28.7 ± 1.2 ^(n)^	42.3 ± 1.3	40.4 ± 0.9	44.1 ± 1.0 ^m^	39.8 ± 1.2 ^n^	***	***	***	***
SGR ^3^	1.6 ± 0.1 ^a^	1.4 ± 0.1 ^b^	1.6 ± 0.0 ^m^	1.5 ± 0.1 ^n^	2.3 ± 0.1 ^B^	2.3 ± 0.0	2.4 ± 0.0 ^A,m^	2.2 ± 0.1 ^n^	***	***	***	***
DFI ^4^	3.2 ± 0.2	3.0 ± 0.2	3.2 ± 0.2	3.2 ± 0.1	4.1 ± 0.2	4.3 ± 0.1	4.1 ± 0.1	4.1 ± 0.2	***	***	***	***
FCR ^5^	2.1 ± 0.2	2.2 ± 0.1	1.9 ± 0.1	2.1 ± 0.1	1.8 ± 0.0 ^(b)^	1.9 ± 0.0 ^(a)^	1.7 ± 0.0	2.0 ± 0.1				
PER ^6^	1.1 ± 0.1	0.9 ± 0.2	1.1 ± 0.1	1.0 ± 0.1	1.2 ± 0.1	1.1 ± 0.1	1.3 ± 0.0	1.1 ± 0.1				
PPV ^7^	16.7 ± 1.8	14.3 ± 3.1	17.1 ± 1.7	15.6 ± 0.8	18.5 ± 1.4	17.7 ± 0.6	19.2 ± 0.8	17.7 ± 1.8		(*)		
HSI ^8^	2.4 ± 0.3	2.7 ± 0.1	2.4 ± 0.1	2.5 ± 0.2	2.0 ± 0.1	2.1 ± 0.2	1.7 ± 0.2	1.9 ± 0.2	***	***	***	***
FCF ^9^	1.6 ± 0.0	1.6 ± 0.0	1.6 ± 0.0	1.6 ± 0.1	1.7 ± 0.0	1.7 ± 0.0	1.7 ± 0.0	1.7 ± 0.0	***	***	***	***
*Nutrient composition (% wet weight)*												
Dry matter	30.9 ± 0.8	29.6 ± 0.6	30.1 ± 0.2 ^(n)^	30.7 ± 0.2 ^(m)^	33.5 ± 1.8	32.4 ± 0.6	33.2 ± 1.6	32.9 ± 1.0		*		
Crude ash	3.8 ± 0.0	3.9 ± 0.1	3.8 ± 0.0	3.9 ± 0.2	3.4 ± 0.1 ^(b)^	3.6 ± 0.1 ^(a)^	3.6 ± 0.1	3.7 ± 0.1	***	**	**	
Crude protein	16.0 ± 0.2	16.2 ± 0.1	15.8 ± 0.5	16.1 ± 0.2	16.0 ± 0.8	16.3 ± 0.1	15.8 ± 0.3	16.5 ± 0.4				
Crude lipid	10.7 ± 0.8	9.2 ± 0.7	10.0 ± 0.2	10.2 ± 0.3	14.1 ± 0.9 ^(a)^	12.4 ± 0.9 ^(b)^	13.6 ± 1.3	12.7 ± 0.6	***	***	***	**
Gross energy ^10^	8.07 ± 0.32	7.44 ± 0.28	7.72 ± 0.02	7.83 ± 0.08	9.31 ± 0.54	8.73 ± 0.31	9.13 ± 0.60	8.86 ± 0.37	**	**	***	*

^1^ IBW = Initial body weight [g]

^2^ FBW = Final body weight [g]

^3^ SGR = Specific growth rate [% d^− 1^]

^4^ DFI = Daily feed intake [% d^− 1^]

^5^ FCR = Feed conversion ratio

^6^ PER = Protein efficiency ratio

^7^ PPV = Protein productive value [%]

^8^ HSI = Hepatosomatic index [%]

^9^ FCF = Fulton condition factor

^10^ Gross energy is given in MJ kg^-1^ OM.

Initial nutrient composition: dry matter: 29.7% wet weight; crude ash: 4.2% wet weight; crude protein: 17.0% wet weight; crude lipid: 8.4% wet weight; gross energy: 7.34 MJ kg^-1^ wet weight. Values (mean ± SD, n = 3; HSI: n = 15, FCF: n = 63) with different superscript letters and different types of letters within one temperature treatment differ with *p*-values < 0.05 based on ANOVA as described in Materials & methods. Superscript letters indicate the output of tests based on comparisons of fish oil level within one supplement group (a, b: F6 vs. F2; m, n: F6 + G vs. F2 + G) and effect of supplementation within one fish oil level group (A, B: F6 vs. F6 + G). The statistical output of tests based on the effect of the different holding temperatures analyzed within one feeding group are indicated in separate columns using

* for p < 0.05

** for p < 0.01, and

*** for p < 0.001. All designations in brackets indicate a tendency towards a difference based on p < 0.1.

**Table 4:** Statistical significance of effects (p-values) caused by dietary fish oil level (Oil), genistein supplementation (G), rearing temperature (Temp.), and the interaction of either two factors or all three factors. P-values are given for effects on selected growth and performance parameters, body nutrients, and fatty acids (FAs) of different tissues of gilthead sea bream at the end of the 8 week trial.

		Single effects						Interactions						
		Oil		G		Temp.		Oil:G		Oil:Temp.		G:Temp.	Oil:G:Temp.	
Growth and performance	FBW ^1^	<0.001	***	0.035	*	<0.001	***	0.241		0.531		0.343	0.133	
	SGR ^2^	<0.001	***	0.033	*	<0.001	***	0.152		0.420		0.369	0.018	*
	Crude lipid	0.007	**	0.924		<0.001	***	0.084	(*)	0.316		0.712	0.446	
	Gross energy	0.034	*	0.983		<0.001	***	0.096	(*)	0.585		0.865	0.476	
Whole body FAs	SFA ^3^	<0.001	***	0.154		<0.001	***	0.388		0.141		0.207	0.766	
	MUFA ^4^	<0.001	***	0.027	*	<0.001	***	0.118		<0.001	***	0.408	0.230	
	PUFA ^5^	0.002	**	0.032	*	<0.001	***	0.143		0.028	*	0.982	0.470	
	EPA + DHA ^6^	<0.001	***	0.153		<0.001	***	0.573		<0.001	***	0.449	0.411	
Liver tissue FAs	SFA	0.054	(*)	0.401		<0.001	***	0.818		0.275		0.183	0.183	
	MUFA	0.005	**	0.580		0.970		0.067	(*)	0.235		0.297	0.618	
	PUFA	0.402		0.911		<0.001	***	0.206		0.196		0.183	0.311	
	EPA + DHA	<0.001	***	0.761		0.006	**	0.009	**	0.286		0.853	0.018	*
Fillet tissue FAs	SFA	<0.001	***	0.044	*	<0.001	***	0.539		0.177		0.639	0.117	
	MUFA	<0.001	***	<0.001	***	0.013	*	0.913		0.582		0.184	0.051	(*)
	PUFA	<0.001	***	<0.001	***	<0.001	***	0.770		0.206		0.429	0.016	*
	EPA + DHA	<0.001	***	0.269		<0.001	***	0.367		0.107		0.910	0.023	*

^1^ FBW = Final body weight

^2^ SGR = Specific growth rate

^3^ SFA = Saturated fatty acids

^4^ MUFA = Monounsaturated fatty acids

^5^ PUFA = Polyunsaturated fatty acids

^6^ EPA + DHA = Sum of eicosapentaenoic acid (EPA) and docosahexaenoic acid (DHA).

Significant effects are indicated with

* (p < 0.5)

** (p < 0.01), and

*** (p < 0.001). A statistical tendency (p < 0.1) is indicated by (*).

At the end of the experimental period, the whole body nutrient composition did not differ significantly between sea bream from different feeding groups compared within one temperature treatment (Table 3). Nevertheless, the holding temperature of 23°C led to significantly increased crude ash, crude lipid, and gross energy in comparison to sea bream held at 19°C (p < 0.05, Table 3, indicated by *).

### Fatty acid composition

#### Fatty acid composition of whole body homogenates was predominantly affected by dietary fish oil level and holding temperature

At the end of the experimental period, the fatty acid composition of sea bream whole body homogenates was mainly affected by the interaction of the dietary fish oil level and the holding temperature (Table 4). Generally, feeding the F6-based diets led to a significantly higher level of several PUFAs (EPA, DHA, and docosapentaenoic acid (DPA; 22:5n-3)) in the fish tissue in comparison to feeding the F2-based diets (p < 0.05, Table 5, indicated by a, b and m, n). The tissue amount of monounsaturated fatty acids (MUFAs) was affected the opposite way with higher values in F2-fed fish (p > 0.05, Table 5, indicated by a, b and m, n). These effects were visible in fish from both holding temperatures, although the difference was often higher in fish held at 23°C. Dietary genistein caused only a slight, but significant, reduction of tissue MUFAs in fish fed with the diet F6 + G in comparison to fish fed with the diet F6 (p < 0.05, Table 5, indicated by A, B). This effect was temperature dependent and only visible in fish held at 19°C.

**Table 5:** Fatty acid composition (percentage of total fatty acid methyl esters (% FAMEs)) of whole body homogenate of gilthead sea bream at the end of the 8 week feeding trial held at two different temperatures (19°C and 23°C). F6 and F2 indicate the feeding with the control diets containing 6% and 2% DM fish oil, respectively. +G indicates feeding diets supplemented with 0.15% DM genistein.

		19°C				23°C				Comparison between 19 and 23°C			
*% FAMEs*	Initial	F6	F2	F6 + G	F2 + G	F6	F2	F6 + G	F2 + G	F6	F2	F6 + G	F2 + G
14:0	4.2	2.8 ± 0.1 ^a^	2.5 ± 0.1 ^b^	2.8 ± 0.1 ^m^	2.5 ± 0.1 ^n^	2.8 ± 0.1 ^a^	2.3 ± 0.1 ^b^	2.7 ± 0.0 ^m^	2.3 ± 0.0 ^n^		*		*
16:0	17.8	17.2 ± 0.2	16.8 ± 0.2	17.0 ± 0.2	16.9 ± 0.2	18.9 ± 0.2	18.6 ± 0.5	18.8 ± 0.2	18.4 ± 0.0	***	***	***	***
18:0	4.2	4.4 ± 0.2	4.6 ± 0.1	4.4 ± 0.1	4.6 ± 0.1	5.1 ± 0.2	4.9 ± 0.1	4.8 ± 0.1	4.9 ± 0.2	***	*	**	*
∑ SFA ^1^	26.3	24.4 ± 0.4	23.9 ± 0.2	24.3 ± 0.1	23.9 ± 0.2	26.9 ± 0.4 ^a^	25.9 ± 0.5 ^b^	26.3 ± 0.3 ^(m)^	25.6 ± 0.2 ^(n)^	***	***	***	***
14:1n-5	0.1	0.1 ± 0.0	0.1 ± 0.0	0.1 ± 0.0	0.1 ± 0.0	0.1 ± 0.0	0.1 ± 0.0	0.1 ± 0.0	0.1 ± 0.0		*		
16:1n-7	6.2	4.4 ± 0.2 ^a^	3.9 ± 0.1 ^b^	4.4 ± 0.0 ^m^	3.9 ± 0.1 ^n^	4.5 ± 0.1 ^a^	3.7 ± 0.2 ^b^	4.5 ± 0.1 ^m^	3.6 ± 0.1 ^n^				(*)
18:1n-7c	3.8	3.1 ± 0.1 ^a^	3.0 ± 0.0 ^b^	3.2 ± 0.0 ^m^	3.0 ± 0.1 ^n^	2.9 ± 0.1 ^a^	2.6 ± 0.0 ^b^	2.8 ± 0.0 ^m^	2.6 ± 0.1 ^n^	***	***	***	***
18:1n-9c	28.5	32.0 ± 0.7 ^A^	32.8 ± 0.3	30.6 ± 0.1 ^Bn^	32.7 ± 0.6 ^m^	31.6 ± 0.4 ^b^	35.5 ± 0.4 ^a^	31.4 ± 0.5 ^n^	35.3 ± 0.5 ^m^		***		***
∑ MUFA ^2^	38.5	39.5 ± 0.9 ^A^	39.7 ± 0.2	38.2 ± 0.1 ^Bn^	39.6 ± 0.7 ^m^	39.2 ± 0.1 ^b^	41.8 ± 0.6 ^a^	38.8 ± 0.6 ^n^	41.6 ± 0.4 ^m^		***		**
18:2n-6c	13.5	18.8 ± 0.6	19.5 ± 0.2	19.3 ± 0.1	19.5 ± 0.3	17.7 ± 0.3 ^(b)^	18.6 ± 0.7 ^(a)^	18.3 ± 0.4	18.8 ± 0.2	*	(*)	*	
18:3n-3	3.0	7.5 ± 0.3	7.6 ± 0.2	7.7 ± 0.1	7.7 ± 0.1	7.4 ± 0.0 ^b^	8.0 ± 0.4 ^a^	7.8 ± 0.2	8.1 ± 0.2				(*)
18:3n-6	0.4	0.9 ± 0.1 ^b^	1.2 ± 0.0 ^a^	1.0 ± 0.1	1.1 ± 0.1	0.8 ± 0.0	0.9 ± 0.1	0.8 ± 0.1	1.0 ± 0.1		***	*	*
20:5n-3	6.6	3.0 ± 0.1 ^a^	2.7 ± 0.2 ^b^	3.2 ± 0.2 ^m^	2.8 ± 0.2 ^n^	2.9 ± 0.1 ^a^	1.7 ± 0.0 ^b^	2.9 ± 0.1 ^m^	1.7 ± 0.1 ^n^		***	*	***
22:5n-3	2.8	1.3 ± 0.1 ^a^	1.2 ± 0.1 ^b^	1.3 ± 0.1 ^m^	1.1 ± 0.0 ^n^	1.1 ± 0.0 ^a^	0.7 ± 0.0 ^b^	1.0 ± 0.1 ^m^	0.7 ± 0.0 ^n^	***	***	***	***
22:6n-3	9.0	4.6 ± 0.2	4.2 ± 0.3	4.9 ± 0.2 ^m^	4.3 ± 0.4 ^n^	4.0 ± 0.0 ^a^	2.5 ± 0.1 ^b^	4.1 ± 0.2 ^m^	2.6 ± 0.1 ^n^	*	***	**	***
∑ PUFA ^3^	35.2	36.1 ± 1.1	36.4 ± 0.4	37.5 ± 0.2	36.4 ± 0.8	34.0 ± 0.3 ^(a)^	32.3 ± 1.1 ^(b)^	34.9 ± 0.9 ^m^	32.8 ± 0.6 ^n^	*	***	**	***
EPA + DHA ^4^	15.6	7.7 ± 0.3 ^(a)^	6.9 ± 0.5 ^(b)^	8.2 ± 0.4 ^m^	7.1 ± 0.6 ^n^	6.9 ± 0.1 ^a^	4.1 ± 0.1 ^b^	7.0 ± 0.3 ^m^	4.3 ± 0.2 ^n^	(*)	***	**	***
EPA/DHA	0.7	0.7 ± 0.0	0.6 ± 0.0	0.7 ± 0.0	0.6 ± 0.0	0.7 ± 0.0	0.7 ± 0.0	0.7 ± 0.0	0.7 ± 0.0	**	*	*	
n-3/n-6	1.5	0.8 ± 0.0	0.8 ± 0.0^b^	0.9 ± 0.0 ^m^	0.8 ± 0.0 ^n^	0.8 ± 0.0 ^a^	0.7 ± 0.0 ^b^	0.8 ± 0.0 ^m^	0.7 ± 0.0 ^n^		***		***

^1^ ∑ SFA is the sum of saturated fatty acids

^2^ ∑ MUFA is the sum of monounsaturated fatty acids

^3^ ∑ PUFA is the sum of n-3 and n-6 polyunsaturated fatty acids

^4^ EPA + DHA is the sum of eicosapentaenoic acid (EPA; 20:5n-3) and docosahexaenoic acid (DHA; 22:6n-3).

Values (mean ± SD, n = 3 (consisting of five individuals per triplicate)) with different superscript letters and different types of letters within one temperature treatment differ with *p*-values < 0.05 based on ANOVA as described in Materials & methods. Superscript letters indicate the output of tests based on comparisons of fish oil level within one supplement group (a, b: F6 vs. F2; m, n: F6 + G vs. F2 + G) and effect of supplementation within one fish oil level (A, B: F6 vs. F6 + G). The statistical output of tests based on the effect of the different holding temperatures analyzed within one feeding group are indicated in separate columns using

* for p < 0.05

** for p < 0.01 and

*** for p < 0.001. All designations in brackets indicate a tendency towards a difference based on p < 0.1.

#### Fatty acid composition of liver tissue responded to the interaction of all three factors (dietary fish oil level, genistein, and holding temperature)

At the end of the experiment, the fatty acid composition of liver tissue of sea bream was mainly affected by temperature and dietary treatment. The amount of saturated fatty acids (SFAs) was higher in fish held at the higher water temperature whereas single MUFAs were rather affected by the dietary fish oil level (Table 4). A reduction of dietary fish oil from 6% DM (diet F6) to 2% DM (diet F2) led to significant reductions in tissue EPA, DPA and DHA levels (p < 0.05, Table 6, indicated by a, b and m, n). This effect could be observed in fish held at both temperatures. Additionally, the amounts of longer chained fatty acids (> C20) responded to the presence of dietary genistein. Genistein led to a decrease of EPA, DPA and DHA in fish fed the diet F2 + G in comparison to fish fed the diet F2 (p < 0.05, Table 6, indicated by M, N). On the contrary, an increase in the tissue EPA content was visible in fish fed the diet F6 + G in comparison to fish fed diet F6 (p < 0.05, Table 6, indicated by A, B). These adverse effects were temperature dependent and only visible in fish held at 19°C, indicating that the interaction of all three factors (Table 4) is needed to influence the sum of liver EPA + DHA contents.

**Table 6:** Fatty acid composition (percentage of total fatty acid methyl esters (% FAMEs)) of liver tissue of gilthead sea bream at the end of the 8 week feeding trial held at two different temperatures (19°C and 23°C). F6 and F2 indicate the feeding with the control diets containing 6% and 2% DM fish oil, respectively. +G indicates feeding diets supplemented with 0.15% DM genistein.

		19°C				23°C				Comparison between 19 and 23°C			
*% FAMEs*	Initial	F6	F2	F6 + G	F2 + G	F6	F2	F6 + G	F2 + G	F6	F2	F6 + G	F2 + G
14:0	2.0	1.7 ± 0.1	1.7 ± 0.1	1.6 ± 0.3	1.6 ± 0.2	2.2 ± 0.3	2.2 ± 0.2	1.9 ± 0.2	1.9 ± 0.2	*	*		
16:0	16.0	15.5 ± 0.6	14.1 ± 0.8	15.0 ± 1.3	15.5 ± 1.3	19.4 ± 0.8	18.4 ± 0.4	19.2 ± 1.0 ^(m)^	17.1 ± 1.5 ^(n)^	**	***	***	
18:0	8.6	6.5 ± 0.5	6.4 ± 0.4	6.4 ± 0.4	6.3 ± 0.3	7.8 ± 0.0	7.6 ± 0.2	7.7 ± 0.4	7.2 ± 0.4	**	**	**	*
∑ SFA ^1^	26.5	23.6 ± 1.0	22.2 ± 1.2	23.0 ± 1.8	23.4 ± 1.8	29.4 ± 0.9	28.2 ± 0.7	28.7 ± 1.2	26.2 ± 2.0	***	***	***	
14:1n-5	0.0	0.0 ± 0.0	0.0 ± 0.0	0.1 ± 0.0	0.0 ± 0.0	0.0 ± 0.0	0.0 ± 0.0	0.0 ± 0.0	0.0 ± 0.0			(*)	
16:1n-7	3.7	3.8 ± 0.1 ^a^	3.1 ± 0.1 ^b^	4.2 ± 0.4 ^m^	3.2 ± 0.3 ^n^	3.8 ± 0.1 ^a^	3.2 ± 0.2 ^b^	3.6 ± 0.1 ^(m)^	3.1 ± 0.4 ^(n)^			*	
18:1n-7c	4.6	3.0 ± 0.1	2.9 ± 0.0	3.0 ± 0.1	3.0 ± 0.0	2.8 ± 0.1 ^B(a)^	2.7 ± 0.1 ^(b)^	3.1 ± 0.1 ^Am^	2.6 ± 0.1 ^n^	(*)	**		***
18:1n-9c	30.7	39.0 ± 0.7	41.1 ± 0.5	38.0 ± 2.1 ^n^	43.6 ± 2.7 ^m^	40.3 ± 1.9	41.5 ± 0.3	39.0 ± 1.0 ^(n)^	42.3 ± 1.0 ^(m)^				
∑ MUFA ^2^	39.0	45.8 ± 0.6	47.1 ± 0.4	45.3 ± 2.3 ^n^	49.9 ± 2.9 ^m^	47.0 ± 1.9	47.4 ± 0.4	45.7 ± 1.1	48.0 ± 1.4				
18:2n-6c	7.6	16.2 ± 0.8	16.3 ± 1.2	15.9 ± 1.5	15.3 ± 2.8	11.9 ± 0.5	13.1 ± 0.6	12.6 ± 0.5	14.2 ± 1.6	**	*	*	
18:3n-3	1.5	5.9 ± 0.5	5.3 ± 0.5	5.6 ± 0.7	5.0 ± 1.2	4.2 ± 0.3	4.3 ± 0.4	4.2 ± 0.2	5.0 ± 0.7	*	(*)		
18:3n-6	1.6	2.2 ± 0.2 ^b^	3.7 ± 0.9 ^a^	2.1 ± 0.4	3.3 ± 0.9	1.9 ± 0.1 ^b^	3.5 ± 0.6 ^a^	3.1 ± 0.8	3.2 ± 0.9				
20:5n-3	7.2	2.0 ± 0.1 ^B^	1.7 ± 0.4 ^M^	2.7 ± 0.5 ^Am^	0.9 ± 0.2 ^Nn^	1.8 ± 0.1 ^a^	1.1 ± 0.1 ^b^	1.9 ± 0.2 ^m^	1.0 ± 0.1 ^n^		(*)	*	
22:5n-3	3.8	0.7 ± 0.0	0.5 ± 0.1 ^M^	0.8 ± 0.1 ^m^	0.3 ± 0.1 ^Nn^	0.6 ± 0.1 ^a^	0.3 ± 0.0 ^b^	0.6 ± 0.1 ^m^	0.4 ± 0.0 ^n^		*	(*)	
22:6n-3	12.9	3.6 ± 0.3	3.2 ± 0.8 ^M^	4.7 ± 1.1 ^m^	1.9 ± 0.4 ^Nn^	3.2 ± 0.2	2.1 ± 0.3	3.2 ± 0.5 ^(m)^	2.0 ± 0.2 ^(n)^		(*)	*	
∑ PUFA ^3^	34.5	30.5 ± 1.6	30.7 ± 1.4	31.7 ± 4.1	26.7 ± 4.3	23.6 ± 1.1	24.4 ± 1.1	25.5 ± 2.1	25.8 ± 3.4	*	*	*	
EPA + DHA ^4^	20.0	5.6 ± 0.4 ^(B)^	4.9 ± 1.2 ^M^	7.4 ± 1.6 ^(A)m^	2.8 ± 0.6 ^Nn^	5.0 ± 0.3 ^(a)^	3.2 ± 0.5 ^(b)^	5.1 ± 0.6 ^m^	3.1 ± 0.3 ^n^		(*)	*	
EPA/DHA	0.6	0.6 ± 0.0	0.5 ± 0.0	0.6 ± 0.0 ^m^	0.5 ± 0.0 ^n^	0.6 ± 0.0	0.5 ± 0.0	0.6 ± 0.0 ^m^	0.5 ± 0.0 ^n^				
n-3/n-6	2.8	0.7 ± 0.0 ^Ba^	0.5 ± 0.1 ^Mb^	0.8 ± 0.1 ^Am^	0.4 ± 0.1 ^Nn^	0.7 ± 0.0 ^a^	0.5 ± 0.0 ^b^	0.6 ± 0.0 ^m^	0.5 ± 0.0 ^n^			**	

^1^ ∑ SFA is the sum of saturated fatty acids

^2^ ∑ MUFA is the sum of monounsaturated fatty acids

^3^ ∑ PUFA is the sum of n-3 and n-6 polyunsaturated fatty acids

^4^ EPA + DHA is the sum of eicosapentaenoic acid (EPA; 20:5n-3) and docosahexaenoic acid (DHA; 22:6n-3).

Values (mean ± SD, n = 3 (consisting of samples from five individuals per triplicate)) with different superscript letters and different types of letters within one temperature treatment differ with *p*-values < 0.05 based on ANOVA as described in Materials & methods. Superscript letters indicate the output of tests based on comparisons of fish oil level within one supplement group (a, b: F6 vs. F2; m, n: F6 + G vs. F2 + G) and effect of supplementation within one fish oil level (A, B: F6 vs. F6 + G; M, N: F2 vs. F2 + G). The statistical output of tests based on the effect of different holding temperatures analyzed within one feeding group are indicated in separate columns using

* for p < 0.05

** for p < 0.01 and

*** for p < 0.001. All designations in brackets indicate a tendency towards a difference based on p < 0.1.

#### Fatty acid composition of fillet tissue can be affected by the interaction of all three factors (dietary fish oil level, genistein, and holding temperature)

After the experimental period, the fatty acid composition of the fillet tissue of sea bream was affected by the interaction of all three factors: dietary fish oil level, dietary genistein, and holding temperature (Table 4). The dietary fish oil level likewise affected SFAs, MUFAs and PUFAs of fish from all dietary treatments and both holding temperatures (Table 7). Feeding of the F6-based diets led to significantly decreased SFAs and some MUFAs, whereas the fatty acid 18:1n-9 was increased, same as the sum of all MUFAs (p < 0.05, Table 7, indicated by a, b and m, n). The amounts of EPA, DPA, DHA and the sum of all PUFAs decreased with decreasing dietary fish oil level, namely feeding of the F2-based diets (p < 0.05, Table 7, indicated by a, b and m, n). These effects of the dietary fish oil level were independent from the holding temperature. Generally, the higher holding temperature of 23°C led to higher levels of SFAs and lower levels of most PUFAs, whereas only single MUFAs were affected (Table 7, p-values indicated by *).

**Table 7:** Fatty acid composition (percentage of total fatty acid methyl esters (% FAMEs)) of fillet tissue of gilthead sea bream at the end of the 8 week feeding trial held at two different temperatures (19°C and 23°C). F6 and F2 indicate the feeding with the control diets containing 6% and 2% DM fish oil, respectively. +G indicates feeding diets supplemented with 0.15% DM genistein.

		19°C				23°C				Comparison between 19 and 23°C			
*% FAMEs*	Initial	F6	F2	F6 + G	F2 + G	F6	F2	F6 + G	F2 + G	F6	F2	F6 + G	F2 + G
14:0	3.4	2.5 ± 0.2 ^a^	2.2 ± 0.2 ^b^	2.6 ± 0.1 ^m^	2.3 ± 0.1 ^n^	2.6 ± 0.1 ^a^	2.1 ± 0.0 ^b^	2.5 ± 0.1 ^m^	2.0 ± 0.2 ^n^				*
16:0	17.8	17.9 ± 0.1 ^a^	17.2 ± 0.2 ^b^	17.5 ± 0.0	17.2 ± 0.2	19.2 ± 0.3	19.5 ± 0.3 ^(M)^	19.4 ± 0.2	19.0 ± 0.3 ^(N)^	***	***	***	***
18:0	4.2	4.3 ± 0.2	4.4 ± 0.1	4.3 ± 0.2	4.3 ± 0.1	4.9 ± 0.2	4.9 ± 0.2	4.7 ± 0.1	4.9 ± 0.1	***	**	*	***
∑ SFA ^1^	25.4	24.8 ± 0.2 ^a^	23.8 ± 0.3 ^b^	24.4 ± 0.1 ^(m)^	23.8 ± 0.3 ^(n)^	26.7 ± 0.3	26.5 ± 0.4 ^(M)^	26.6 ± 0.2 ^m^	25.9 ± 0.5 ^(N)n^	***	***	***	***
14:1n-5	0.1	0.1 ± 0.0	0.1 ± 0.0	0.1 ± 0.0	0.0 ± 0.0	0.1 ± 0.0	0.1 ± 0.0	0.1 ± 0.0	0.1 ± 0.0			***	**
16:1n-7	5.1	4.0 ± 0.3 ^a^	3.6 ± 0.3 ^b^	4.1 ± 0.1 ^m^	3.5 ± 0.1 ^n^	4.2 ± 0.1 ^a^	3.6 ± 0.1 ^b^	4.2 ± 0.1 ^m^	3.4 ± 0.2 ^n^				
18:1n-7c	3.4	2.9 ± 0.0 ^a^	2.8 ± 0.1 ^b^	3.0 ± 0.0 ^m^	2.8 ± 0.1 ^n^	2.7 ± 0.1 ^a^	2.4 ± 0.1 ^b^	2.8 ± 0.0 ^m^	2.4 ± 0.0 ^n^	**	***	***	***
18:1n-9c	29.4	31.2 ± 0.5 ^Ab^	33.0 ± 0.3 ^a^	30.0 ± 0.1 ^Bn^	32.6 ± 0.4 ^m^	31.1 ± 0.2 ^b^	34.0 ± 0.7 ^a^	30.9 ± 0.4 ^n^	33.5 ± 0.6 ^m^		(*)	*	(*)
∑ MUFA ^2^	37.9	38.2 ± 0.1 ^Ab^	39.4 ± 0.3 ^a^	37.0 ± 0.1 ^Bn^	38.9 ± 0.3 ^m^	38.0 ± 0.2 ^b^	40.0 ± 0.7 ^(M)a^	37.9 ± 0.5 ^m^	39.3 ± 0.5 ^(N)n^			*	
18:2n-6c	14.6	19.7 ± 0.2	20.1 ± 0.5	20.1 ± 0.3	20.6 ± 0.3	18.5 ± 0.1 ^(b)^	19.1 ± 0.2 ^(N)(a)^	18.7 ± 0.0 ^n^	19.8 ± 0.4 ^(M)m^	***	**	***	*
18:3n-3	4.2	8.3 ± 0.1	8.3 ± 0.2 ^N^	8.5 ± 0.3	8.8 ± 0.2 ^M^	7.8 ± 0.1	8.2 ± 0.2	8.0 ± 0.2	8.2 ± 0.3	(*)		*	*
18:3n-6	0.4	0.8 ± 0.0 ^b^	0.9 ± 0.1 ^a^	0.7 ± 0.0 ^n^	1.0 ± 0.1 ^m^	0.7 ± 0.0	0.7 ± 0.1	0.7 ± 0.1	0.8 ± 0.1		***		**
20:5n-3	5.4	2.8 ± 0.1	2.5 ± 0.2	3.1 ± 0.1 ^m^	2.3 ± 0.1 ^n^	2.7 ± 0.0 ^a^	1.8 ± 0.3 ^b^	2.8 ± 0.2 ^m^	2.0 ± 0.2 ^n^		**		
22:5n-3	2.5	1.2 ± 0.0 ^B^	1.1 ± 0.1	1.4 ± 0.1 ^Am^	1.0 ± 0.0 ^n^	1.2 ± 0.1 ^a^	0.8 ± 0.1 ^b^	1.1 ± 0.1 ^m^	0.8 ± 0.1 ^n^		***	**	**
22:6n-3	9.7	4.3 ± 0.1 ^(B)^	3.9 ± 0.3	4.9 ± 0.3 ^(A)m^	3.6 ± 0.0 ^n^	4.4 ± 0.0 ^a^	3.0 ± 0.4 ^b^	4.3 ± 0.5 ^m^	3.3 ± 0.3 ^n^		**	(*)	
∑ PUFA ^3^	36.7	37.0 ± 0.2 ^B^	36.8 ± 0.5	38.6 ± 0.1 ^Am^	37.4 ± 0.5 ^n^	35.3 ± 0.2 ^a^	33.5 ± 0.9 ^Nb^	35.5 ± 0.6	34.9 ± 0.4 ^M^	**	***	***	***
EPA + DHA ^4^	15.1	7.1 ± 0.2	6.3 ± 0.4	8.0 ± 0.4 ^m^	5.9 ± 0.1 ^n^	7.1 ± 0.1 ^a^	4.8 ± 0.7 ^b^	7.0 ± 0.7 ^m^	5.3 ± 0.5 ^n^		**	(*)	
EPA/DHA	0.6	0.7 ± 0.0	0.6 ± 0.0	0.6 ± 0.0	0.6 ± 0.0	0.6 ± 0.0	0.6 ± 0.0	0.6 ± 0.0	0.6 ± 0.0				
n-3/n-6	1.4	0.8 ± 0.0 ^(B)a^	0.8 ± 0.0 ^b^	0.9 ± 0.02 ^(A)m^	0.7 ± 0.0 ^n^	0.8 ± 0.0 ^a^	0.7 ± 0.0 ^b^	0.8 ± 0.0 ^m^	0.7 ± 0.0 ^n^		(*)		

^1^ ∑ SFA is the sum of saturated fatty acids

^2^ ∑ MUFA is the sum of monounsaturated fatty acids

^3^ ∑ PUFA is the sum of n-3 and n-6 polyunsaturated fatty acids

^4^ EPA + DHA is the sum of eicosapentaenoic acid (EPA; 20:5n-3) and docosahexaenoic acid (DHA; 22:6n-3).

Values (mean ± SD, n = 3 (consisting of samples from five individuals per triplicate)) with different superscript letters and different types of letters within one temperature treatment differ with *p*-values < 0.05 based on ANOVA as described in Materials & methods. Superscript letters indicate the output of tests based on comparisons of fish oil level within one supplement group (a, b: F6 vs. F2; m, n: F6 + G vs. F2 + G) and effect of supplementation within one fish oil level (A, B: F6 vs. F6 + G; M, N: F2 vs. F2 + G). The statistical output of tests based on the effect of the different holding temperatures analyzed within one feeding group are indicated in separate columns using

* for p < 0.05

** for p < 0.01 and

*** for p < 0.001. All designations in brackets indicate a tendency towards a difference based on p < 0.1.

The dietary supplementation of genistein led to changes in the fatty acid composition almost exclusively of fish held at 19°C. The fatty acid 18:1n-9 and the sum of MUFAs were decreased in fish fed the diet F6 + G in comparison to the diet F6 (p < 0.05, Table 7, indicated by A, B). On the contrary, fillet DPA amounts and the sum of PUFAs were significantly increased by 16.7% and 4.3%, respectively, in fish fed the genistein-supplemented diet F6 + G in comparison to the diet F6 (p < 0.05, Table 7, indicated by A, B). Furthermore, the amount of DHA and the n-3/n-6 ratio were improved in tendency by the supplementation of genistein in comparison to the F6 diet (p < 0.1, Table 7, indicated by (A), (B)).

## Discussion

### Growth performance and nutrient composition

The gilthead sea bream is a fast-growing warm-water species and the growth observed within this study matches this trait. The SGR was comparable to or better than the 1.2–1.9%^-d^ reported by other studies [45,46]. A higher water temperature led to an increased feed intake (DFI: 4.1–4.3%^-d^) and thus promoted growth (SGR and FBW). This was expected and had previously been reported for warm-water species like the gilthead sea bream and the European sea bass [35,47]. Contrasting, the feed conversion of all groups (FCR: 1.7–2.2) was poorer compared to other studies with sea bream of similar size [45,46]. A systemic effect, namely lost pellets due to the water current in the cylindrical tanks, might have led to an underestimation of the FCR. This has also resulted in low PER (0.9–1.2) and PPV (14.3–19.2%) values when compared to the aforementioned studies. Additionally, the diets containing only 5% DM fish meal and 2 or 6% DM fish oil might have impaired the nutrient utilization of sea bream. To the best of our knowledge, such low inclusion levels of marine ingredients in diets for sea bream have only been used in the previous study from our lab [48], where similar performance was recorded. Thus, it cannot be clearly distinguished whether a systemic effect, the diet formulation, or their combination led to an overall effect on the nutrient utilization parameters. The diet formulation did not affect the whole body nutrient composition significantly. Solely temperature led to differences, which might in return be effects of increased feed intake and better growth rates of fish held at the higher temperature. The elevated lipid content of sea bream held at the higher water temperature is in accordance with similar findings in sea bass [35,47]. Overall, temperature had the most distinctive effect on the investigated growth performance and nutrient composition. The three-factorial study design was only proven useful to slightly increase the growth rates: The combination of the higher temperature, a dietary fish oil level of 6% DM, and genistein supplementation led to the highest SGR in this study.

It is noteworthy that dietary genistein had no further effect on the growth performance and especially lipid content of sea bream in this experiment. Soy isoflavones like genistein are described to exhibit lipid lowering effects in several mammalian species like rats, mice, and rabbits [18,24,26,49]. Furthermore, the metabolism and utilization of lipids and gross energy can be affected by dietary genistein and other soy isoflavones in some fish species [29,50,51]. Studies from our group with rainbow trout revealed that genistein did not affect fish growth at dietary concentrations of 0.15–0.30% DM [41,52]. Nevertheless, the high inclusion of 0.30% DM genistein in the diet led to impaired nutrient digestibility, especially crude lipid and gross energy in rainbow trout [51], whereas a lower concentration of 0.15% DM had no effect on the body composition of rainbow trout [52]. It is generally accepted that depending on the concentration, the effects of genistein might even be the opposite. In line with the previous studies, we deduce that the concentration of genistein (0.15% DM) and its dietary application had no effect on growth performance and nutrient composition of sea bream in this experiment.

### Fatty acid composition

The fatty acid composition of sea bream usually reflects that of its diet [45,53,54]. Deviating from that, the fatty acid composition of sea bream from this trial was modified by three factors: dietary fish oil level, dietary genistein, and the holding temperature. In sea bream, tissue SFA and MUFA contents can be modified effectively by the choice of dietary oil source [45,55]. Terrestrial plant oils contain considerable amounts of SFAs and MUFAs, for example palmitic acid (16.0) and oleic acid (18:1n-9), which are absent in fish oils [56–58]. In this study, the sea bream fed the diet containing only 2% DM fish oil and thus larger amounts of plant oils, had an increased whole body MUFA content dominated by the fatty acid 18:1n-9, whereas the SFA content remained largely unaffected. This indicates a difference in the incorporation of SFAs and MUFAs into the different fish tissues that is not solely mediated by the dietary amount of these FAs, but also by other factors. Low water temperatures would favor a shift in the SFA/MUFA ratio towards MUFAs to maintain physiological and cellular functions and membrane fluidity [33]. In this trial, the unchanged amounts of MUFAs but decreased amounts of SFAs at the lower temperature support this finding. This indicates a temperature-mediated modification of MUFA and SFA levels on top of effects caused by their dietary levels.

Supporting the initial hypothesis, the reduced dietary fish oil level of 2% DM led to decreased EPA, DPA, DHA and PUFA levels in all investigated tissues of sea bream. This effect was more pronounced when the sea bream were held at the higher water temperature of 23°C. With regard to the initial samples, a general reduction of PUFAs took place during the experimental period, more pronounced in fish held at 23°C. Since the experimental diets had lower n-3 LC-PUFA levels than the commercial diet fed during the adaptation phase, the common phenomenon of fish oil wash-out and dilution of tissue fatty acids had occurred [55,59]. Most likely, the fast growth and thus higher tissue turnover of sea bream held at 23°C accelerated this process, leading to more pronounced effects of the fish oil wash-out in these groups. A study with sea bream reported a combined effect of increased water temperature and reduced dietary fish oil as reason for reduced tissue n-3 fatty acid levels [47]. Furthermore, it is likely that lower water temperatures counteract the n-3 PUFA wash-out and favor the incorporation of these fatty acids into membranes to maintain the physiological functionality of cells [5,35,60].

Interestingly, the effect of dietary genistein on the fatty acid composition of sea bream was dependent on the dietary fish oil level and the holding temperature. Furthermore, the different fish tissues reacted differently towards the single stimuli, revealing an interesting insight into approaches targeting an improvement of the fillet lipid quality. Whereas the whole body fatty acid composition remained largely unaffected by dietary genistein, clear effects of genistein were visible in the fillet and liver tissue. Thus, dietary genistein improved the fillet tissue retention of n-3 LC-PUFAs in sea bream held at 19°C and fed diets containing 6% DM fish oil. In the livers of corresponding fish, the same effects were visible, too, although they did not reach statistical significance. This was most likely due to the large variation of the values within one feeding group. In both tissues, the retention was affected by the holding temperature, apparently via reduced growth rates and thus lowered metabolism and consequently fatty acid wash-out in seabream held at the lower temperature. The question on how genistein improved the tissue fatty acid retention remains to be elucidated and can only be hypothesized at this point. It is thinkable that antioxidant properties of genistein protected the LC-PUFAs from oxidation in the tissue [30]. Cell culture studies have indicated, that the antioxidant activity of genistein can be mediated by its free-radical scavenging properties [61]. Furthermore, genistein activated the Nrf1 transcription factor in endothelial cells, thereby increasing the expression of glutathione peroxidase, an enzyme responsible for cellular antioxidant defence mechanisms [62]. Nevertheless, it is indispensable for this mode of action that considerable amounts of genistein reach the fillet and liver tissue. Since the bioavailability of genistein is regarded to be rather low [63], such antioxidant effects might only play a minor role, especially in the fillet. Other regulatory mechanism targeting genes encoding for enzymes involved in the fatty acid synthesis are feasible in sea bream [8,64]. Such mechanisms could be related to estrogen-like effects of genistein. Genistein is a phytoestrogen, and has great structural similarity to the estrogen 17β-estradiol [65] and can thus bind to estrogen receptors. The hormone estrogen is reported to be the reason for a more effective fatty acid synthesis resulting in increased DHA tissue levels in females compared to males, as shown in humans by Giltay et al. [66] and in rats by Extier et al. [67]. The underlying modes of action seem to be a direct influence of estrogen on the enzymes involved in the LC-PUFA production, namely fatty acyl desaturases and elongases [67–69]. It is thus that genistein may affect the same pathways as the hormone estrogen. In fish, namely all-female rainbow trout, genistein led to elevated tissue DHA levels [41,52]. Especially in marine fish, where the first part of the fatty acid synthesis (production of EPA from ALA) is disrupted but the synthesis of DHA from EPA seems to be functional [70–72], genistein might lead to an increase in tissue DHA levels. This is partly supported by the results from this study. Furthermore, juvenile sea bream are exclusively male (protandrous hermaphrodites) and thus, genistein might act differently than in female counterparts. Another possible mode of action could be a transcriptional regulation of the peroxisomal β-oxidation via the transcription factor PPARα and its target genes, which are responsive to genistein [21,25,32]. In the present investigation, the fatty acid composition of the liver tissue of genistein-fed sea bream partly matches the aforementioned findings. Genistein led to an increase in the amount of EPA + DHA when the sea bream were fed the diet with 6% DM fish oil + genistein, but simultaneously led to a decrease when the low fish oil diet supplemented with genistein was fed. Such difference in the response of the sea bream to dietary genistein cannot be explained solely by genistein, but seems to depend on the dietary fish oil level, too. It is widely accepted that EPA and DHA can affect the fatty acid synthesis by a negative feedback loop (reviewed in Nakamura and Nara [73]). Furthermore, dietary lipids are generally reflected in the fatty acid composition of fish tissues [45]. It seems likely that the combination of the different modes of action of genistein and the effects of dietary fatty acids on the fatty acid synthesis and tissue composition have led to the current observations in sea bream. This was supported by the significant combined effect proven for temperature, dietary fish oil level, and dietary genistein on the liver and fillet fatty acid composition.

## Conclusion

The use of genistein in diets for juvenile gilthead sea bream had neither a clear beneficial, nor a detrimental effect on fish growth performance and whole body nutrient composition. Whereas the whole body fatty acid composition was rather affected by the combination of dietary fish oil level and temperature, the liver and fillet tissue fatty acid composition was affected by the combination of all three factors (dietary fish oil level, dietary genistein, temperature). Furthermore, the dietary fish oil level and thus the varying dietary fatty acid compositions were of importance for the response of the tissues towards genistein. The temperature most likely had an overall effect on sea bream via altered growth rates and fatty acid metabolism. It can be concluded that although the effects of genistein on the fatty acid composition of sea bream were only marginal, the three-factorial approach was proven useful to improve fillet and liver fatty acid composition. It would be highly interesting to deepen the investigations on a molecular level, especially in order to highlight the combined effects of dietary fish oil and genistein on the genetic ability of sea bream to perform fatty acid synthesis.
